# Comprehensive RNA-seq transcriptomic profiling in the malignant progression of gliomas

**DOI:** 10.1038/sdata.2017.24

**Published:** 2017-03-14

**Authors:** Zheng Zhao, Fanlin Meng, Wen Wang, Zheng Wang, Chuanbao Zhang, Tao Jiang

**Affiliations:** 1 Beijing Neurosurgical Institute, Beijing 100050, China; 2 School of Medicine, Tsinghua University, Beijing 100084, China; 3 Department of Neurosurgery, Beijing Tiantan Hospital, Capital Medical University, Beijing 100050, China; 4 Department of Neurosurgery, The Second Affiliated Hospital of Soochow University, Suzhou 215123, China; 5 Centre of Brain Tumour, Beijing Institute for Brain Disorders, Beijing 100069, China; 6 China National Clinical Research Centre for Neurological Diseases, Beijing 100050, China

**Keywords:** CNS cancer, RNA sequencing, Transcriptomics

## Abstract

Gliomas are the most common and lethal intracranial tumours. RNA sequencing technologies and advanced data analyses recently enabled the characterization of transcriptomic information, including protein-coding gene expression, non-coding gene expression, alternative splicing, and fusion gene detection, to facilitate detection of diseases and altered phenotypes. As a part of the Chinese Glioma Genome Atlas (CGGA) project, our aim was to delineate comprehensive transcriptome profiling in the malignant progression of human gliomas. Three hundred twenty five gliomas with different grades were collected over the past twelve years. Using the Illumina HiSeq 2,000 system, over 92 million high quality 101-bp paired-end reads were generated per sample, yielding a total of 30 billion reads. This comprehensive dataset will be useful to deepen the comprehensive understanding of gliomas, providing an opportunity to generate new therapies, diagnoses, and preventive strategies.

## Background & Summary

Malignant gliomas are the most common and lethal primary brain tumours. According to the multicentre cross-sectional study on brain tumours (MCSBT) in China, age standardized prevalence of primary brain tumours is approximately 22.52 per million for all populations, and that of gliomas is 31.1% in those aged 20–59 years^1^. Although there have been advances in the current standard of care, the survival for patients with gliomas has still remained unchanged, especially for aggressive gliomas^2,3^. High-throughput whole-transcriptome sequencing technologies, i.e., RNA-seq, have the capability to capture the transcriptomic landscape with an unprecedented high-resolution. RNA-seq technology is able to generate comprehensive transcriptomic information at different levels, including quantification of protein-coding and non-coding gene expression, identification of non-coding RNAs (ncRNAs) and/or fusion genes, and determination of alternative splicing^4,5^. Using RNA-seq to catalog abnormal events in the malignant progression of gliomas, from low-grade to high-grade dynamic range, can provide insights into disease susceptibility and identification of biomarkers, and could improve the translation of basic medicine to clinical therapy.

As a part of the Chinese Glioma Genome Atlas (CGGA, http://cgga.org.cn/) project, an RNA-seq profiling dataset for Chinese glioma patient cohort was generated to identify oncogenic fusions associated with glioma progression as previously described^5^. We revealed a novel, recurrent PTPRZ1-MET (ZM) fusion transcript in secondary glioblastomas, which was independently validated in other groups^6^. By combining this dataset with paired protein data, we also indicated that enhanced MET oncoprotein expression and phosphorylation were induced by glioma-specific ZM fusions^7^. To the best of our knowledge, this dataset represent the largest public RNA-seq data on Chinese glioma patient cohort. The principal aim of this study was to provide a comprehensive resource to deposit the raw RNA-seq data sets underpinning the advanced studies on dynamic transcriptomic signatures with the malignant progression of human gliomas. To this end, a total of three hundred and twenty five clinical samples were collected from several hospitals in China. These samples were chosen as they represented glioma patients with different grades of disease progression, allowing comparison of low-grade samples to their high-grade counterparts. The histopathological grades for these samples were as follows: 109 samples of grade II, 72 samples of grade III, and 144 samples of grade IV. As one of the deadliest cancers, grade IV glioma, termed glioblastoma (GBM), has a poor median survival time of only 12 to 18 months^2,8–10^. Although most GBMs are primary, approximately twenty percent of them are secondary from the progression of previous low-grade gliomas of grade II or III^11^. Therefore, it is becoming an urgent clinical task to identify early biomarkers for diagnosis and prognosis, as well as to explore the mechanisms underlying the development and progression of gliomas. This data set will therefore be of important and indispensable values for the glioma research community at large.

In this work, the RNA-seq libraries were sequenced using the Illumina HiSeq 2,000 platform. Approximately 30 billion 101-bp paired-end RNA-seq reads were generated, with an average of over 92 million sequence reads per sample. For each sample, we first aligned the raw reads to the genome using MapSplice (v2.1.7)^12^ and annotated the reads to RefSeq, resulting in 17,527 genes. Next, we developed a web-based, user-friendly human glioma transcriptomic profiling database (GLIOMASdb, http://cgga.org.cn:9091/gliomasdb/) to deposit and catalog the transcriptomic profiles of the cohort of 325 glioma samples. In a few words, we have provided a comprehensive RNA-seq data resource on the malignant progression of Chinese glioma, which is useful to understand the progression of the disease. Furthermore, the data resource provides an opportunity to identify the biomarkers of early warning, diagnosis and prognosis, which have the potential to be applied to clinical treatments of glioma.

## Methods

### Clinical specimen collection

A total of 325 glioma samples were obtained from several hospitals in China from 2004 to 2016. All research performed was approved by the Tiantan Hospital Institutional Review Board (IRB) and kept consistent with the principles of the Helsinki Declaration. All the subjects were diagnosed with gliomas by consensus, according to central pathology reviews by independent board-certified neuropathologists and further graded based on the 2007 WHO classification. Written informed consent was obtained from all patients. The specimens were collected under IRB KY2013-017-01 and were frozen in liquid nitrogen within 5 min of resection. The following clinical information of each patient was also collected: age, sex, diagnosis, WHO grade, chemoradiotherapy and epilepsy therapy status, etc. (Table 1).

### RNA-seq library preparation and sequencing

**Figure 1:**
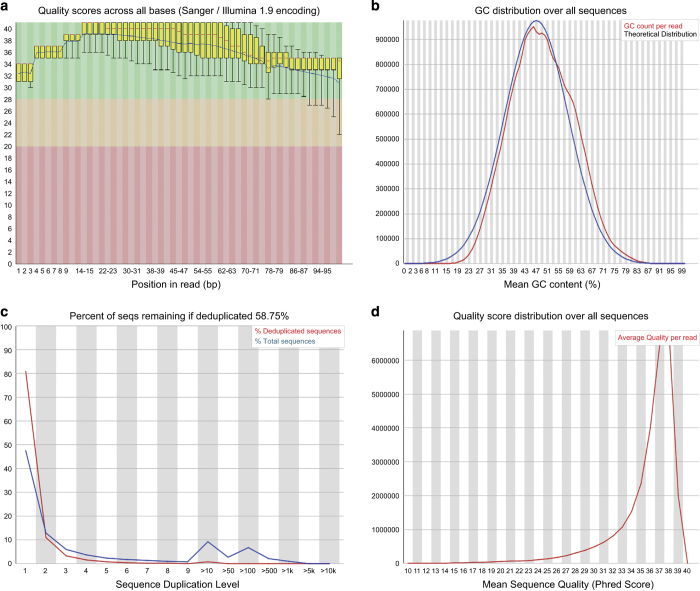
A representative example of quality control metrics of RNA sequenced reads as indicated by FastQC (sample: CGGA_909). (**a**) Phred quality score distribution over all reads in each base. (**b**) GC content (%) distribution over all sequences. (**c**) The distribution of duplicated reads compared to the total number of sequences. (**d**) Quality score distribution over all sequences.

Prior to library preparation, total RNA was isolated using RNeasy Mini Kit (Qiagen) according to the manufacturer’s instructions. Pestle and QIAshredder (Qiagen) were used to disrupt and homogenize frozen tissue. The RNA intensity was checked using a 2,100 Bioanalyzer (Agilent Technologies) and only high quality samples with an RNA Integrity Number (RIN) value greater than or equal to 6.8 were used to construct the sequencing library. Typically, 1 μg of total RNA was used with the TruSeq RNA library preparation kit (Illumina) in accordance with low-throughput protocol, except that SuperScript III reverse transcriptase (Invitrogen) was used to synthesize first strand cDNA. After PCR enrichment and purification of adapter-ligated fragments, the concentration of DNA with adapters was determined with quantitative PCR (Applied Biosystems 7,500) using primers QP1 5′-AATGATACGGCGACCACCGA-3′ and QP2 5′-CAAGCAGAAGACGGCATACGAGA-3′. The length of the DNA fragment was measured using a 2,100 Bioanalyzer, with median insert sizes of 200 nucleotides. Then, RNA-seq libraries were sequenced using the Illumina HiSeq 2,000 Sequencing System. The libraries were prepared using the paired-end strategy with the read length of 101 bp. Base-calling was performed by the Illumina CASAVA v1.8.2 pipeline. The quality of the RNA-seq libraries was first evaluated using the FastQC v0.11.5 software (http://www.bioinformatics.bbsrc.ac.uk/projects/fastqc/) (Fig. 1). Then, the reads were subjected to standard quality control (QC) criteria according to the following parameters: (1) reads that aligned to primers and/or adaptors with no more than two mismatches, (2) reads with over 50% of low-quality bases (quality value ≤5) in one read, and (3) reads with over 10% unknown bases (N bases). After QC, over 30 billion filtered reads were used for further analysis.

### RNA-seq data analysis

RNA-seq mapping and quantification were processed according to the pipeline in our previous research^7^. RNA-seq data from 325 grade II-IV gliomas was obtained from the Chinese Glioma Genome Atlas (CGGA) project. Applying the same TCGA mRNA-seq pipeline (University of North California RNA-seq workflow), expression analysis was performed with modifications. First, clean reads were aligned to the human genome reference (hg19) with MapSplice (v2.1.7)^12^, followed by BAM file sorting with SAMtools (v0.1.9)^13^. Second, sequencing read counts for each RefSeq gene were calculated using RSEM (v1.2.15)^14^. Third, expression levels from different samples were normalized by the Trimmed Mean of M values (TMM) method^15^. Finally, the normalized expression levels of different samples were merged into a FPKM (fragments per kilobase transcriptome per million fragments) matrix for further analysis.

Differential expression analyses were performed to reveal critical genes associated with the malignant progression of gliomas by comparing gene expression in pairwise grades of gliomas. A gene was defined to be associated with the malignant progression of gliomas only if it presented differential expression in the comparison of any two grades. It was required for the genes of differential expression to satisfy both *P*-value and fold-change criteria simultaneously (|log2(Fold change)|≥1 and FDR<0.05). The *P*-values were obtained using the unpaired Student’s *t*-test and the false discovery rate (FDR) was controlled by the Benjamini and Hochberg algorithm.

### A database for human gliomas on RNA-seq

To facilitate community-wide use of this unique RNA-seq data on the malignant progression of gliomas, we have developed a free, web accessible, user-friendly transcriptomic profiling database (GLIOMASdb, http://cgga.org.cn:9091/gliomasdb/) for Chinese gliomas. Here, users can easily explore the expression patterns of gene of interest (e.g., mRNA or lncRNA) in the malignant progression of gliomas using the BROWSE interface. In addition, GLIOMASdb currently provides data download including gene expression profiles and matched phenotypic data.

### Code availability

The code is available on the *Figshare* (Data Citation 1).

## Data Records

The raw fastq files for the RNA-seq data from the 325 gliomas in different stages of malignant progression, as part of the CGGA data sets, have been deposited in the SRA website (NCBI-SRA) under accession numbers SRP027383 (Data Citation 2) and SRP091303 (Data Citation 3). In addition, the complete datasets, including expression profiles (Data Citation 1) and matched clinical information (Data Citation 1), are available on the *Figshare* or downloaded from the GLIOMASdb web site: http://cgga.org.cn:9091/gliomasdb/.

## Technical Validation

### Quality control—RNA integrity

To determine the quality of the RNA-seq libraries, RNA intensity was checked using a 2,100 Bioanalyzer (Agilent Technologies). The integrity of the total RNA was calculated by the RNA Integrity Number (RIN) algorithm, which can be used to estimate RNA quality and degradation level. Higher RIN values indicate better integrity of total RNA, with the highest RIN value being 10. In this study, only total RNAs with a RIN value of greater than or equal to 6.8 were used to construct the sequencing library, which shows RNA with high integrity was used.

### Quality validation and analyses

We applied FastQC v0.11.5 software to determine data quality and analysed several measures, including the sequence quality per base, GC content per sequence, sequence duplication levels, and quality score distribution over all sequences of the fastq files. A representative summary plot is depicted (CGGA_909). Here, the quality scores per base were high, with a median quality score above 30 suggesting high quality sequences across all bases (Fig. 1a). The GC distribution per base over all sequences was examined. The GC composition pattern was similar to theoretical distribution indicating that the samples were contaminant free (Fig. 1b). In addition, sequence complexity was examined. Approximately 15% of sequences were shown over 10 to 100 times, either from highly expressed transcripts duplications (Fig. 1c). The quality score distribution over all sequences was analysed to see if a subset of sequences had universally poor quality. The average quality for most sequences was high, with scores above 37, which indicated that a significant proportion of the sequences in a run had overall high quality (Fig. 1d). All other fastqc files were shown to have similar quality metrics compared to sample CGGA_909.

**Figure 2:**
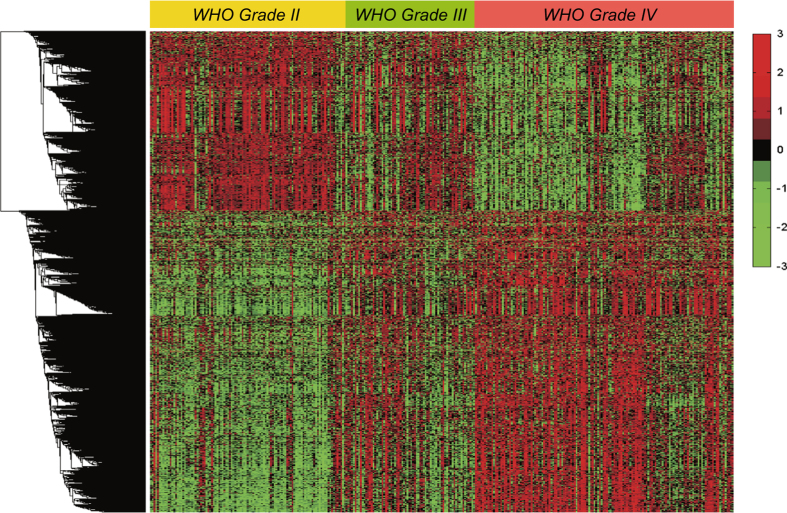
Heatmap and one-dimensional hierarchical clustering of differentially expressed genes (DEGs) across different grades of gliomas. The genes are displayed in rows and samples are displayed in columns.

The aim of this study was to depict the comprehensive RNA-Seq transcriptomic profiling of Chinese gliomas. For the analyses we chose samples from grade II-IV gliomas based on central pathology reviews by independent board-certified neuropathologists and the 2007 WHO classification. All are human gliomas, differing in molecular pathology, primarily in terms of malignant progression and possible genetic background. Using a heatmap depicting differentially expressed genes using one-dimensional hierarchical clustering analysis, one can intuitively observe that samples along the malignant progression gradient showed different expression patterns (Fig. 2). Previously, Cheng *et al.* ^16^ showed eight immune-related genes (FOXO3, IL6, IL10, ZBTB16, CCL18, AIMP1, FCGR2B, and MMP9) with the greatest prognostic value in grade IV gliomas. Most of which (6/7, no data for FCGR2B in this study) we confirmed were differentially expressed between different grades of gliomas.

## Usage Notes

A wide range of public software, e.g., MapSplice^12^, TopHat^17^, RSEM^14^, Cufflinks^18^, could be used for transcript assembly and quantification. Other public software combinations could be efficiently used for quantification: e.g., HISATs^19,20^ and StringTie^21^ for quantification, used together with the ballgown package^19,22^ for differential expression analyses. Currently, several gene annotations are available, such as RefSeq, UCSC, and Ensembl. With the rapid development of RNA-seq technology, we also recommend use of the more comprehensive gene annotation generated from the GENCODE^23^ project, that reveals many new classes of RNAs, including pseudogenes and different types of ncRNAs.

A major advantage of this study is that we aimed to comprehensively profile the RNA-seq transcriptome along the malignant progression of gliomas. Consistent with previous research, we identified a set of differentially expressed genes involved in gliomas that may be biomarkers and therapeutic targets. However, there are several existing classifications for human gliomas, including Kernohan, AANS and excellent WHO grade classification. Although these classifications are able to simply recognize the malignant degree of gliomas, they cannot accurately determine the prognosis of patients and are not suitable for the current clinical treatments. We expect that other classification criterion will be developed based on the molecular pathology for understanding gliomas and precise medicines.

## Additional Information

**How to cite this article:** Zhao, Z. *et al.* Comprehensive RNA-seq transcriptomic profiling in the malignant progression of gliomas. *Sci. Data* 4:170024 doi: 10.1038/sdata.2017.24 (2017).

**Publisher’s note:** Springer Nature remains neutral with regard to jurisdictional claims in published maps and institutional affiliations.

## Supplementary Material



## Figures and Tables

**Table 1:** Patient Characteristics.

	**All Patients (** ***N*** **=325)**	
**Characteristics**	**No.**	**%**
	*Age classes*	
<18	3	0.92
18–35	71	21.85
36–39	51	15.69
40–49	103	31.69
50–59	61	18.77
60–69	31	9.54
≥70	5	1.54
	*Sex*	
Male	203	62.46
Female	122	37.54
	*WHO grade classes*	
Grade II	109	33.54
Grade III	72	22.15
Grade IV	144	44.31
	*TCGA classes*	
Classical	74	22.77
Mesenchymal	68	20.92
Neural	81	24.92
Proneural	102	31.39
	*Chemoradiotherapy*	
Radiotherapy plus other drugs	8	2.46
Radiotherapy plus temozolomide	78	24.00
Radiotherapy plus unknown drug	64	19.69
Chemotherapy with temozolomide alone	12	3.69
Chemotherapy with unknown drug alone	14	4.31
Radiotherapy alone	87	26.77
No data for radiotherapy plus temozolomide	1	0.31
Radiotherapy plus no data for chemotherapy	2	0.62
No data for chemoradiotherapy	27	8.31
No therapy	32	9.84
	*Epilepsy therapy*	
Anti-epileptic	85	26.15
No therapy	157	48.31
No data	83	25.54
